# Effect of the degree of follicular diameter ≥18mm differentiation on the day of hCG administration to the outcome of controlled ovarian hyperstimulation (COH)

**DOI:** 10.3389/fendo.2024.1414213

**Published:** 2024-06-28

**Authors:** Hongyi Xu, Qi Chen, Jiarong Tian, Xin Chen, Xin Zhang, Xin Li, Ying Wu, Changjun Zhang, Ying Zhang

**Affiliations:** ^1^ Reproductive Medicine Center, Renmin Hospital, Hubei University of Medicine, Shiyan, China; ^2^ Hubei Clinical Research Center for Reproductive Medicine, Shiyan, China; ^3^ Biomedical Engineering College, Hubei University of Medicine, Shiyan, China; ^4^ Biomedical Research Institute, Hubei University of Medicine, Shiyan, China; ^5^ Hubei Key Laboratory of Embryonic Stem Cell Research, Hubei University of Medicine, Shiyan, China; ^6^ Department of Medical Laboratory, Renmin Hospital, Hubei University of Medicine, Shiyan, China

**Keywords:** follicular differentiation, standard deviation, human chorionic gonadotropin trigger, controlled ovarian hyperstimulation, pregnancy outcomes

## Abstract

**Objective:**

To explore the impact of the level of differentiation in a minimum of two follicles with a diameter of ≥18 mm on the outcome of controlled ovarian hyperstimulation on the day of human chorionic gonadotropin (hCG) administration.

**Methods:**

Single-center data from January 2018 to December 2021 was retrospectively analyzed for 1,199 patients with fresh embryo transfer for assisted reproduction. The absolute value of the standard deviation of the follicle size of at least 2 follicles ≥18 mm in diameter in both ovaries on the day of hCG was taken as the degree of differentiation of the dominant follicle after ovulation induction, based on the standard deviation response to the degree of dispersion of the data. The degree of follicular differentiation was divided into 3 groups according to the size of the value, and the general clinical conditions, laboratory indexes, and clinical outcomes of the patients in the 3 groups were compared.

**Results:**

Among the three groups, the body mass index (BMI) of the ≤1s group was lower than that of the other two groups (P< 0.05), while the follicle-stimulating hormone (FSH) and Anti-Mullerian hormone (AMH) were higher (P< 0.05), and the implantation rate and clinical pregnancy rate were significantly higher than those of the other two groups (P< 0.01). After multifactorial logistic regression to correct for confounding factors, with the ≤1s group as the reference, the implantation rate, hCG-positive rate, clinical pregnancy rate and live birth rate of embryo transfer in the ≥2S group were significantly lower (P< 0.01). The results of curve fitting analysis showed that the live birth rate decreased gradually with the increase of the absolute standard deviation (P=0.0079).

**Conclusion:**

Differences in follicle diameters ≥18 mm on the day of hCG injection did not have an impact on embryo quality, but had an impact on pregnancy outcomes. The less the variation in follicle size, the more homogeneous the follicle development and the higher the likelihood of live births.

## Introduction

Controlled ovarian hyperstimulation (COH) in assisted reproductive technology is designed to achieve better live birth outcomes (1). The quality of the oocytes is a prerequisite for better outcomes, and clinicians’ assessment of oocyte quality is primarily based on criteria such as the morphology of oocyte-corona-cumulus complexs (OCCCS) and follicle diameter (2). The diameter of the follicle can predict the maturity of the oocyte (3), and only after reaching a certain diameter can it complete its maturation and development to the MII stage (4, 5), thus obtain more efficient fertilization and embryo quality (6). In order to obtain superior quality mature oocytes, the doctor injects hCG at the best time during ovulation induction according to hormone level, follicle size (7, 8).

The size of the dominant follicle diameter is one of the most significant indicators for predicting the quality and developmental potential of most oocytes and embryos (9). It has been reported that hCG injections are generally performed when at least three dominant follicles reach >16 mm (10), or at least three ≥ 17 mm (11) or at least two ≥ 18 mm (12, 13). Oocytes obtained from these dominant follicle populations have a higher embryonic development potential than mature oocytes extracted from small follicles (10). However, in practice, follicle development is not necessarily synchronized in the dominant follicle group. There are few studies on follicle homogeneity, and most of them are studies on animals, such as horses (14), cattle (15, 16), rats (17), etc. Most reports of follicular homogeneity in humans are mainly based on the ratio of large follicles to small follicles (10, 18) or the synchronicity of follicle waves (15, 19), but there is no suitable indicator to reflect the homogeneity of follicle diameter on hCG days.

In this study, at least two follicles with a diameter of ≥18mm were taken as the research object on the day of hCG injection, and the difference between the standard partial absolute value of the dominant follicle diameter and size was used as the differentiation degree of follicles, and its effect on pregnancy outcome was further explored, so as to provide a certain basis and practical plan for the treatment of COH in assisted reproduction.

## Materials and methods

### Study subjects and grouping

The study complied with the Helsinki Declaration basic principles, and all subjects signed the relevant informed consent form before ovulation promotion. Patients with ovulation promotion protocols since 2018 were selected from the standard population: at least 2 dominant follicles with a diameter greater than 18 mm bilaterally on the ovulation promotion trigger day, follicle diameters between 18mm and 30mm were included in the dominant follicle cohort. The degree of dispersion of the response data was expressed as standard deviation, and the standard deviation (s) of follicle diameter for each patient was calculated according to the statistical formula = sqrt(((x1-x) ^2 +(x2-x) ^2 +…… (xn-x) ^2)/(n-1)). One standard deviation absolute value is expressed in 1s. Larger values represent greater dispersion of the data, indicating poorer follicle size consistency. All standard deviations calculated are in the range 0- ± 5.8. Three groups were categorized according to the size of the absolute value of standard deviation, ≤1s group (n=370 cases), 1s-2s group (n=694 cases), and ≥2s group (n=135 cases). Inclusion criteria: (1) the pregnancy-assisting programs were all pituitary down-regulation programs; (2) chromosomally normal for both men and women; (3) no hyperprolactinemia, diabetes mellitus, endocrine diseases with abnormal thyroid function, and systemic diseases of liver and kidney function; (4) no organic lesions of the uterine cavity, and no uterine malformations; (5) at least two follicles ≥18 mm in diameter on hCG day. A total of 2,627 patients were analyzed in the study, of which 1,199 completed the entire process of assisted reproduction and 1428 were not included in the study due to personal reasons and missing data.

### Controlled ovarian hyperstimulation and embryo transfer

3.75 mg long term downregulation plan was used: Ultrasound (Diagnostic ultrasound machine, Aloka SSD-900, Japan) and serum of anti-mullerian hormone (AMH), follicle-stimulating hormone (FSH), luteinizing hormone (LH), estrogen (E2), and progesterone (P) tests were performed on day 3 of menstruation, and 3.75 mg long term dalfylline (Decapeptyl; Ferring, SaintPrex, Switzerland) intramuscularly, and ultrasound and serum sex hormone tests were performed after 30–42 days, and exogenous recombinant gonadotropins (rFSH, Goonafine Merck Serono, Germany) were initiated with 75–300 IU/day or human menopausal gonadotropin (HMG, Zhuhai Lizhu) when E2 was<50 pg/ml, LH<5 mIU/ml, FSH<5 mIU/ml, P<1 ng/ml, follicle diameter of sinus of both ovaries ≤5 mm, and endometrial thickness of the uterine lining ≤5 mm. Ultrasound and serum sex hormone examination were performed on the 4th day of Gn, and the dosage of Gn was adjusted individually according to the results of the examination. Ultrasound and serum FSH, LH, E2, and P examination were performed every 2 days thereafter, and when the follicle diameter reached 16 mm, daily ultrasound and serum FSH, LH, E2 and P examination were started. When at least 2 dominant follicles reached 18mm, hCG 1000–2000 IU (HMG, Zhuhai Lizhu)1 and Aizawa 250 ug were injected, and follicles ≥18 mm were counted and recorded at the same time. Oocyte were pick up by transvaginal ultrasound-guided transvaginal puncture after 34–36 h.

The OCCCs were incubated for 2–4 h after oocyte pick up (OPU). Optimized sperm were added to the culture drop where the OCCCs were located at a sperm concentration of 5000–10000 sperm per OCCCs. Sperm and OCCCs were incubated together for 4 h-5 h, and then the periplasmic granulosa cells were removed. The oocytes were examined under a stereomicroscope to see if the second polar body was discharged. If all the oocytes did not have the second dipolar body discharge or the rate of the second dipolar body discharge was less than 25%, the incubation was continued for half an hour, and if the situation was still the same, then it was judged that there was a failure of fertilization, and rescue Intra-Cytoplasmic Single Sperm Injection (rICSI) was used, and the prokaryotic nuclei were observed overnight, and if two pronucleus (2PN) was observed, then fertilization was considered to have been successful.

Embryos scored on day 3 with 6–8 cleavage spheres, uniform size, regular shape, intact zona pellucida; homogeneous and clear cytoplasm; and ≤20% intraembryonic debris were considered high quality embryos. All embryos continued to be cultured into blastocysts, and those with high scores should be preferred at transfer. This is the Schoolcraft and Gardner scoring system on day 5. High quality blastocysts were defined as stage 3 and above with inner cell mass and trophectoderm scores not containing D or (and) C. The number of embryos to be transferred was strictly determined in accordance with the requirements of the Code of Assisted Human Reproductive Technology, and the total number of embryos to be transferred in each cycle should not be more than 3, of which the number of embryos to be transferred in the first cycle of assisted conception for women under 35 years old should not be more than 2. Ultrasound-guided intrauterine transfer is used. On the 14th day after embryo transfer, a serum hCG test will be performed, and a value greater than 10IU/L is considered positive for hCG, and the presence of a fetal heartbeat on ultrasound examination in the 4th week can be recognized as a clinical pregnancy. A normal child born at more than 28 weeks is considered a live birth.

### Statistical methods

SPSS 26.0 (IBM, Armonk, NY, USA) was used for statistical analysis. Normally distributed continuous variables were expressed as mean ± standard deviation (xˉ ± s). Comparisons between groups were made by one-way analysis of variance (ANOVA), and categorical variables were described by numbers and percentages, and were compared by Pearson’s chi-square test. One-way analyses were used to identify confounding factors affecting clinical outcomes, and multifactorial logistic regression analyses were used to investigate the relationship between differentiation indices and clinical outcomes after adjusting for confounding factors, and adjustment variables were screened for covariance. Smooth curve fitting was used to fit the linear relationship between differentiation indices and live birth rate, combined with smooth curve fitting to analyze the effect of the degree of follicular differentiation on the live birth rate, and the difference was considered statistically significant at P<0.05. Graphs were generated using OriginPro 2018C version 9.5.1.195 (OriginLab).

## Results

### Comparison of general conditions prior to ovulation in patients with different degrees of differentiation

The mean values of BMI and basal follicle-stimulating hormone were significantly different among the three groups (P<0.05), with higher BMI and lower basal follicle-stimulating hormone in the more differentiated patients. The mean values of female age, AMH, basal luteinizing hormone, total Gn use time, total amount used and follicle stimulating hormone on hCG day were not statistically significant between the three groups (P>0.05) (**Table 1**).

**Table 1:** Comparison of general conditions of patients with different differentiation indices.

Item	Follicle differentiation index			F	P
	≤1	1–2	>2		
Female age	28.94 ± 3.56	29.27 ± 3.49	29.44 ± 3.27	1.485	0.227
BMI	22.43 ± 3.75	22.97 ± 3.74	23.32 ± 3.76	3.775	0.023
AMH	7.04 ± 4.22	6.63 ± 4.08	5.95 ± 3.42	3.524	0.030
Basal FSH	6.58 ± 2.74	6.20 ± 1.95	6.10 ± 2.29	4.021	0.018
Basal LH	10.04 ± 12.61	8.61 ± 7.95	8.66 ± 8.90	2.741	0.065
Total duration of Gn used	11.47 ± 1.84	11.45 ± 1.91	11.42 ± 1.78	0.082	0.921
Total dosage of Gn used	2209.65 ± 858.06	2274.44 ± 859.24	2336.74 ± 878.52	1.265	0.283
hCG FSH	14.65 ± 5.64	14.80 ± 5.22	15.34 ± 5.26	0.785	0.456

Date: mean ± SD or (%) (no./total no.). BMI, body mass index; AMH, anti-Mullerian hormone; FSH, follicle-stimulating hormone; LH, luteinizing hormone; IVF, *in vitro* fertilization; ICSI, intracytoplasmic sperm injection; Gn, gonadotropin.

### Cycle outcomes in patients with different differentiation indices

There were no significant differences between infertility factors and technology reproductive adjutant in patients with different levels of differentiation, the normal fertilization rate increased with the increase of differentiation, and there were no statistically significant differences between differentiation in the rates of available embryos, high-quality embryos, and high-quality blastocyst formation, but there were significant differences between different groups in the rates of embryo implantation, HCG positivity, clinical pregnancy, and live births, and all of them decreased with the standard deviation of the decreases with increasing follicle differentiation (P<0.05) (**Table 2**).

**Table 2:** Relationship of different differentiation indices with embryonic parameters and pregnancy outcome.

Item	Follicle differentiation index			χ2	P
	≤1	1-2	>2		
Infertility factor Ovulation failure Pelvic and fallopian tube factors Male factor Unexplained infertility infections Endometriosis Others	77/370(20.81%) 211/370(57.03%) 58/370(15.68%) 10/370(2.70%) 13/370(3.51%) 1/370(0.27%)	176/694(25.36%) 392/694(56.48%) 91/694(13.11%) 16/694(2.31%) 19/694(2.74%) 0	21/135(15.56%) 81/135(60.00%) 25/135(18.52%) 2/135(1.48%) 5/135(3.70%) 1/135(0.74%)	14.108	0.168
Fertilizing style IVF ICSI	303/370(81.89%) 67/370(18.11%)	583/694(84.01%) 111/694(15.99%)	110/135(81.48%) 25/135(18.52%)	1.039	0.595
Normal of fertilization rate (%)	3260/3491(93.38%)	5826/6134(94.98%)	1054/1088(96.88%)	21.713	0.000
Available embryonic rate (%)	1651/3352(49.25%)	2957/6085(48.59%)	523/1110(47.12%)	1.541	0.463
High-quality embryonic rate (%)	1356/1687(80.38%)	2399/3038(78.97%)	429/560(76.61%)	3.803	0.149
High-quality blastocyst formation rate (%)	1310/3375(39.02%)	2368/6147(38.52%)	412/1128(36.52%)	1.961	0.375
Implantation rate (%)	314/461(68.11%)	569/888(64.08%)	96/176(54.55%)	10.214	0.006
Positive hCG rate (%)	282/370(76.22%)	508/694(73.20%)	87/135(64.44%)	6.980	0.030
Clinical pregnancy rate (%)	260/370(70.27%)	458/694(65.99%)	76/135(56.30%)	8.673	0.013
Abortion rate (%)	31/370(8.38%)	66/694(9.51%)	13/135(9.63%)	0.409	0.815
Live birth rate (%)	232/370(62.70%)	401/694(57.78%)	66/135(48.89%)	7.945	0.019

Date: mean ± SD or (%) (no./total no.). BMI, body mass index; AMH, anti-Mullerian hormone; FSH, follicle-stimulating hormone; LH, luteinizing hormone; IVF, *in vitro* fertilization; ICSI, intracytoplasmic sperm injection; Gn, gonadotropin.

### Single factor analysis of live birth rate

The factors that may affect the cumulative live birth rate of patients per cycle after OPU, were analyzed in single factor analysis. The results showed that the age of patients, infertility factors, BMI, and AMH had effects on the live birth rate, and the difference was statistically significant (P<0.05) (**Table 3**).

**Table 3:** Univariate analysis of factors associated with peroocytes retrieval cycle cumulative live birth rate.

Item	Live Birth Rate	
	OR (95%CI)	P
Female age	0.960(0.929-0.992)	0.015
Infertility duration	0.961(0.916-1.008)	0.100
Infertility type Primary infertility Secondary infertility	Ref 1.050(0.834-1.322)	0.679
Infertility factor Pelvic and fallopian tube factors Ovulation failure Male factor Unexplained infertility infections Endometriosis Others	Ref 1.683(1.181-2.121) 1.217(0.867-1.709) 0.693(0.325-1.479) 0.758(0.391-1.469) 0.800(0.050-12.843)	0.002 0.256 0.344 0.412 0.875
BMI	1.038(1.006-1.071)	0.019
AMH	1.044(1.013-1.075)	0.006
Basal Estradiol	0.999(0.997-1.000)	0.088
Basal FSH	1.020(0.969-1.074)	0.442
Basal LH	1.004(0.992-1.016)	0.562
Basal Progestrogen	1.001(0.991-1.012)	0.814
Total duration of Gn used	1	0.155
Total dosage of Gn used	1.046(0.983-1.113)	0.154
No. of oocytes retrieved	1.010(0.963-1.059	0.680

Date: mean ± SD or (%) (no./total no.). BMI, body mass index; AMH, anti-Mullerian hormone; FSH, follicle-stimulating hormone; LH, luteinizing hormone; IVF, *in vitro* fertilization; ICSI, intracytoplasmic sperm injection; Gn, gonadotropin.

### Multivariate logistic regression analysis affecting the cumulative clinical outcomes of each oocyte retrieval cycle

**Figure 1:**
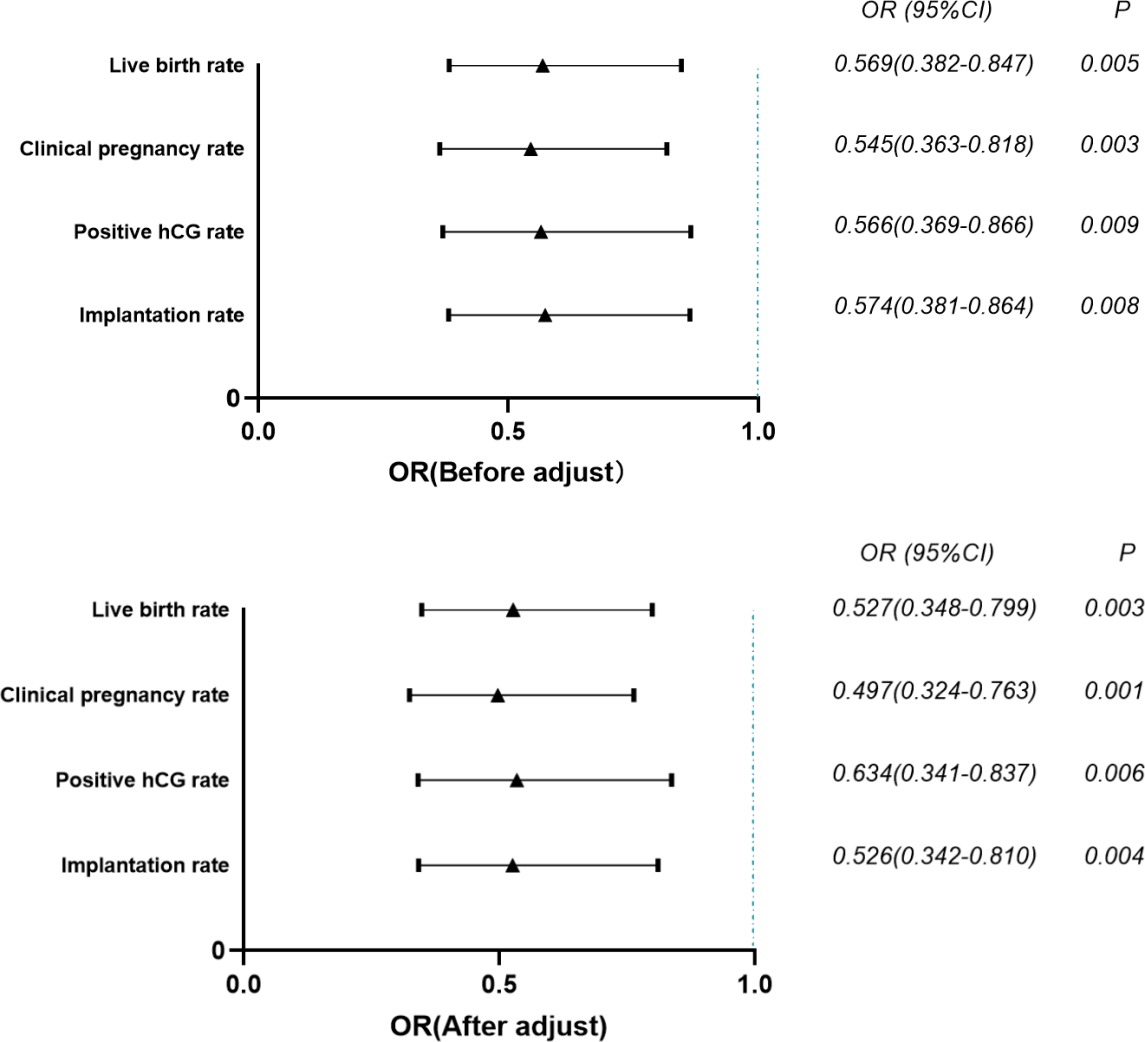
Multivariate logistic regression analysis of the effect of index of differentiation on pregnancy outcomes. p-value < 0.01.

The results of multivariate logistics regression analysis showed that after adjusting for the factors that significantly affected the live birth rate of patients, the embryo implantation rate (OR=0.526, 95% CI=0.342~0.810), positive hCG rate (OR=0.634, 95% CI=0.341~0.837), clinical pregnancy rate (OR=0.497, 95% CI=0.324~0.763), and the live birth rate (OR=0.527, 95% CI=0.348~0.799) of patients’ follicle differentiation ≤ 1s was significantly lower than that > 1s. (P<0.01) (**Figure 1**).

### Curve fitting analysis

**Figure 2:**
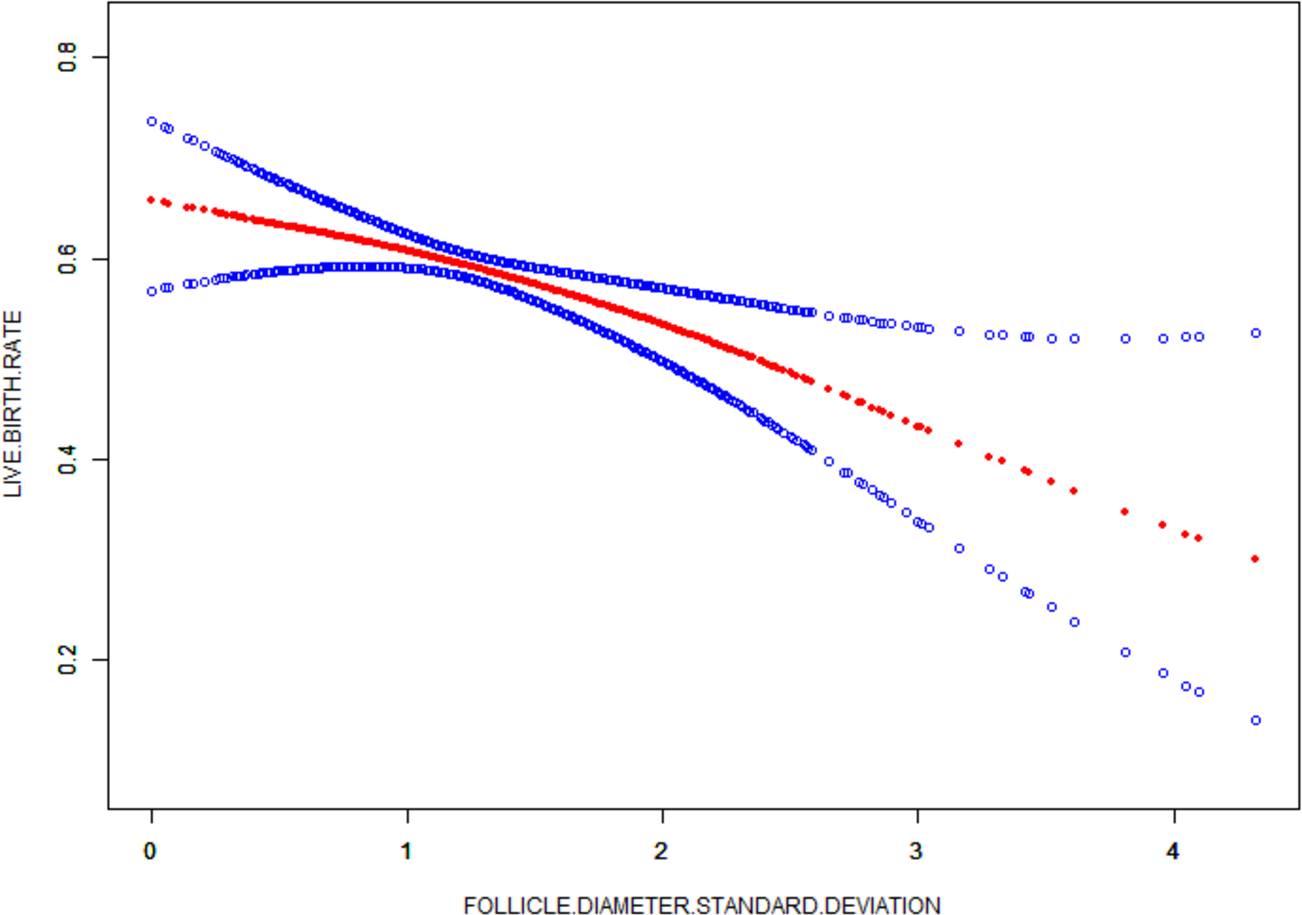
Curve fit of differentiation index to live birth rate. The red curve represents the smooth curve between differentiation index and the live birth rate, the blue curve represents the upper and lower limits of the 95% confidence Interval.

Standard deviation is the degree of dispersion of the data and was used to reflect follicle diameter homogeneity in this study. The curve fitting results of the standard deviation absolute value as the abscissa and the live birth rate on the ordinate showed that the live birth rate increased with the increase of follicle differentiation (P=0.0079) (**Figure 2**).

## Discussion

To the best of our knowledge, this study is the first to reflect follicular synchronization by the standard deviation of follicle diameter size on the day of HCG injection. This study demonstrated that patients in the ≤1s standard deviation of bilateral ovarian follicle diameters ≥18 mm group on the day of HCG injection had lower BMI, exhibited higher AMH and FSH, and had outcomes that showed higher rates of implantation, clinical pregnancy, and live birth. In addition, as the standard deviation increased, the live birth rate of the patients decreased in a linear relationship.

Naturally, one dominant follicle develops and eventually matures under sexual hormone co-regulation, while the other follicles cannot mature and are atrophied and closed (20). In the COH cycle, due to the exogenous use of Gn to increase the FSH concentration, the originally atresia follicles can continue to grow (21), and when multiple follicles grow and develop, the growth rate is variable due to the different responses of each follicle to FSH, which may lead to the production of secondary and tertiary follicles (22), which ultimately leads to a certain difference in follicle diameter, and the difference is further amplified in the later stage of follicle growth, so that some patients have poor follicle homogeneity. There is no consensus in the literature regarding the optimal follicle diameter on hCG day. Most studies have a range of follicle diameters. Studies have reported that hCG injection is initiated when 1–2 dominant follicles reach 18 mm. The larger the dominant follicle diameter, the higher the clinical pregnancy rate (23). Pregnancy rates are highest when hCG is administered in follicles 18–22 mm. In contrast, hCG injections in follicles<17 mm or ≥23 mm have lower pregnancy rates (24). In patients undergoing IUI cycles for ovulatory dysfunction or unexplained infertility, hCG administration at a follicle size of 21.1–22.0 mm was associated with higher clinical odds of pregnancy (25). The optimal preovulatory follicle diameter associated with increased pregnancy rates is between 19 and 20 mm (26). Oocytes obtained from larger follicles may be of better quality (24, 27–29), but it has also been shown that intermediate-sized follicles produce a higher number of available embryos (30). In the present study, the >2S group among the three groups had larger diameter follicles, even as high as 30 mm. This resulted in a large difference in diameter between the other follicles, and the final outcome was poor. It also suggests that large follicle diameters may harm pregnancy outcomes. This may due to the fact that oversized follicles do not have synchronized nuclear and cytoplasmic maturation due to faster growth during development, leading to a rapid increase in follicular volume, which results in poor oocyte quality and a lower rate of oocyte maturation, which ultimately leads to a poorer outcome.

Studies on the synchronization of follicular development do not have a suitable index to reflect this. Studies have reported that follicles with a uniform distribution of all sizes from large follicles (≥20 mm) to intermediate follicles (17–20 mm) to small follicles (<17 mm) are considered to be well synchronized, whereas the predominance of large follicles (≥20 mm) and small follicles (<17 mm) is considered to be different (1). Follicle size and developmental synchrony can influence early human embryo development, and follicular growth patterns have an impact on embryo quality and viability, with the highest implantation rates in the group of large follicles with better uniformly distributed synchrony compared to the group of small follicles (1). In patients with discordant follicular development, asynchronous dominant follicles inhibit the growth and development of adjacent small follicles through the secretion of high levels of INH-B and E2 as well as paracrine and autocrine effects, which in turn affects the maturation rate of the oocytes and the pregnancy rate (2, 3). In this study, the standard deviation of the mean values was used to measure the dispersion of the follicle diameter size distribution. The smaller or larger the standard deviation, the less or more these values deviate from the mean, which indicates better or worse follicular homogeneity. In patients with follicular development that is not chimeric, it was concluded that aspiration of large follicles, thereby eliminating their direct inhibition of neighboring follicles through inter-follicular interactions, significantly improves the rate of oocyte maturation, high-quality embryos, and live births in patients (4, 5). It may be due to the decrease in circulating steroid hormone levels after aspiration of steroid-rich follicular fluid. This avoids the desynchronization of endometrial glandular epithelial and mesenchymal development caused by LH surge and premature progesterone elevation and helps to improve the embryo implantation rate (6, 9). Also in the early follicular stage, we synchronized follicular development and increased the number of oocytes recovered by reference to hormone levels and medication (10).

In addition, FSH and AMH are the main indicators for assessing ovarian function, and in general they are physiologically statistically concordant, but in 20%-43% of patients these values may be discordant (31), and when FSH and AMH values are discordant, AMH is a better predictor of live births in IVF patients. In patients with discordant values, lower AMH was associated with lower live births (32). Conversely higher AMH was associated with higher live births (33). The results of the above studies are in agreement with our results and also indicate that higher levels of AMH follicles have better synchronization and ultimately better pregnancy outcomes despite no difference in embryo quality.

### Strengths and limitations

Our findings provide a new index for the determination of follicular homogeneity and the prediction of pregnancy outcome in patients undergoing clinical superovulation, as well as a possible aid to the clinician’s decision in the course of superovulation therapy. It is worth noting that follicular inhomogeneity on the day of hCG injection may be due to heterogeneity at the initial sinus follicle stage, which is further amplified during later growth (34), or lack of homogeneity at later stages of follicle development due to asynchronous growth rates during development. Thus, a limitation of this study is that only the follicles on the day of hCG injection were studied, and the effect of whether the follicles differentiated during development or before COH on the outcome needs to be further explored. In addition, the present study was retrospective, and because we analyzed real-world data, there may have been an imbalance in baseline characteristics between groups. Well-designed multicenter and large-data studies are needed to further confirm our findings.

### Conclusion

The degree of follicular differentiation of follicles ≥18 mm in diameter on the day of hCG injection in COH cycles does not affect embryo quality, and the smaller the degree of follicular differentiation, the better the follicular homogeneity, which may result in a better outcome of live birth.

### Equations

The equations should be inserted in editable format from the equation editor.


$$\begin{array}{c} \text{Follicular differentiation}\\ =\text{sqrt}\left(\left(\left(x1-x\right)\;\hat{\;}2+\left(x2-x\right)\;\hat{\;}2+\text{…… }\left(xn-x\right)\;\hat{\;}2\right)/\left(n-1\right)\right) \end{array}$$

## Data availability statement

The raw data supporting the conclusions of this article will be made available by the authors, without undue reservation.

## Ethics statement

The studies involving humans were approved by Ethics Committee of Renmin Hospital, Hubei University of Medicine (SYRMYY-061). The studies were conducted in accordance with the local legislation and institutional requirements. The participants provided their written informed consent to participate in this study. Written informed consent was obtained from the individual(s) for the publication of any potentially identifiable images or data included in this article.

## Author contributions

HX: Data curation, Writing – original draft. QC: Data curation, Writing – original draft. JT: Methodology, Writing – review & editing. XC: Data curation, Writing – review & editing. XZ: Supervision, Writing – review & editing. XL: Investigation, Writing – review & editing. YW: Formal analysis, Writing – review & editing. CZ: Formal analysis, Writing – review & editing. YZ: Conceptualization, Writing – review & editing.
